# Prurigo pigmentosa following laparoscopic sleeve gastrectomy: a case report

**DOI:** 10.1093/jscr/rjaf128

**Published:** 2025-03-12

**Authors:** Faisal A Almudaiheem, Ahmed Alzahrani, Majed Alanazi, Mohammed Aljabali, Bandar Ali, Abdulaziz Howil

**Affiliations:** Department of General Surgery, Prince Sultan Military Medical City, Riyadh Makkah Al Mukarramah Road, As Sulimaniyah, Riyadh, 12233, Saudi Arabia; Department of General Surgery, Prince Sultan Military Medical City, Riyadh Makkah Al Mukarramah Road, As Sulimaniyah, Riyadh, 12233, Saudi Arabia; Department of General Surgery, Prince Sultan Military Medical City, Riyadh Makkah Al Mukarramah Road, As Sulimaniyah, Riyadh, 12233, Saudi Arabia; Department of General Surgery, Prince Sultan Military Medical City, Riyadh Makkah Al Mukarramah Road, As Sulimaniyah, Riyadh, 12233, Saudi Arabia; Department of General Surgery, Prince Sultan Military Medical City, Riyadh Makkah Al Mukarramah Road, As Sulimaniyah, Riyadh, 12233, Saudi Arabia; Department of General Surgery, Prince Sultan Military Medical City, Riyadh Makkah Al Mukarramah Road, As Sulimaniyah, Riyadh, 12233, Saudi Arabia

**Keywords:** prurigo pigmentosa, bariatric surgery, metabolic disease, laparoscopic sleeve gastrectomy, ketosis

## Abstract

Prurigo pigmentosa (PP) is a rare dermatological condition with an unclear, often idiopathic etiology, but recent evidence points to a potential link between the disease and metabolic changes, particularly those associated with ketosis. It typically presents as a pruritic, erythematous rash, mainly affecting the trunk and neck, and often features hyperpigmented macules and plaques. The onset of PP has been associated with various factors, including fasting, dieting, and certain metabolic disorders. A high index of suspicion is necessary for diagnosis, as its clinical appearance can be easily mistaken for other skin conditions. Treatment usually involves the use of antibiotics like minocycline to reduce inflammation, along with dietary adjustments aimed at correcting the underlying metabolic disturbances. This patient’s presentation strongly suggests a relationship between PP and the metabolic state induced by bariatric surgery, highlighting the importance of considering PP in post-surgical patients.

## Introduction

Prurigo pigmentosa (PP) was first described in 1917, by a Dr. Masaharu Nagashima, a Japanese dermatologist, it typically presents as pruritic erythematous papules, papulovesicles, and vesicles appearing on the back, chest, or neck [1]. While the etiology is unknown, various mechanical, hormonal, and metabolic triggers have been identified, including ketosis [2]. PP was considered a rare inflammatory dermatosis affecting primarily Asian individuals. However, several case reports subsequently showed that the disease is not restricted to those of Asian origin. Large studies on PP in central European individuals [3]. Most cases reported in have been related to ketogenic diets, and rarely due to bariatric surgery [4].

## Case report

Rayah is a 30 years old Saudi female, who presented to our clinic a case of obesity, her body mass index was 41, she has tried multiple ways of losing weight which have failed, after careful history and physical examination and all routine workup have been done, patient was counseled for laparoscopic sleeve gastrectomy. Surgery was performed electively and was uneventful, the patient had a smooth post-operative recovery period and was discharged in a healthy condition on Day 1.

**Figure 1:**
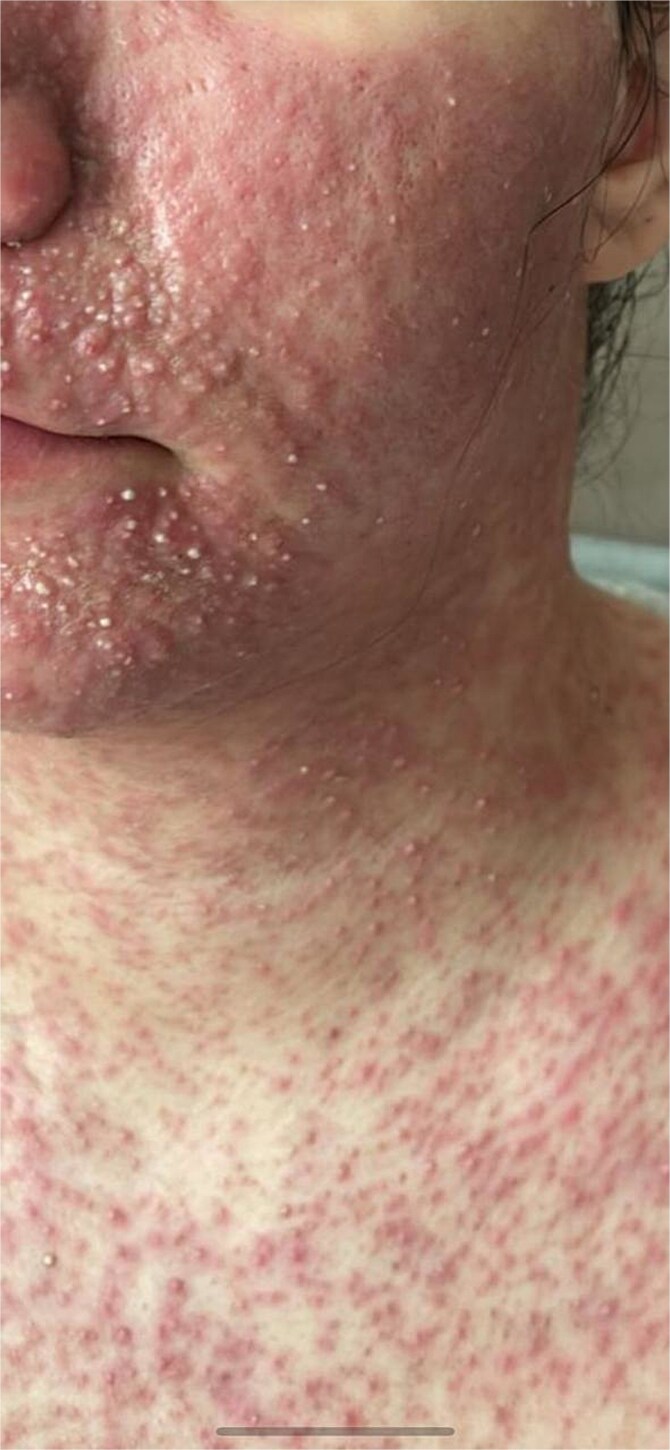
Hyperpigmented patches involving the face and upper trunk.

**Figure 2:**
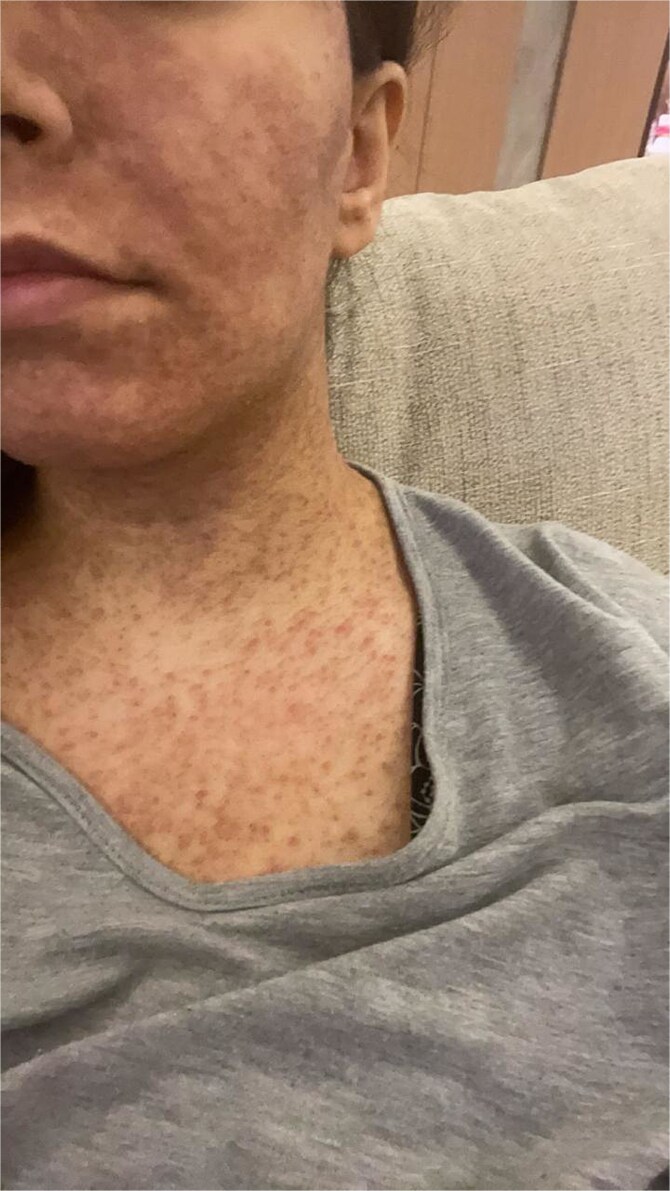
Complete resolution of patient’s rash.

On post-operative Day 6, patient developed a rash on her face and upper body, which required her to visit the dermatology clinic, she has denied deviating from her post-operative diet instructions. On examination the rash is described as multiple reticulated erythematous plaques and reticulated. (Fig. 1), a clinical diagnosis of prurigo pigmentosa was made and antibiotics (minocycline 80 mg) started. Ten days after initiating therapy, patient showed complete resolution of her rash (Fig. 2).

## Discussion

Since Nagashima *et al.* first described it in 1971 [1], over 300 cases of PP have been documented. While it has been predominantly observed in the Japanese population, numerous cases have also been reported in Western countries [3]. With the rise in popularity of the ketogenic diet in recent years, there has been an emergence of PP cases worldwide [2]. The exact pathogenesis of PP remains unclear. However, it has been traditionally linked to various factors, including physical trauma and friction, as well as hormonal influences such as pregnancy and menstruation. Additionally, PP is commonly associated with metabolic disturbances, as previously mention ketotic conditions like dieting, fasting, and diabetes mellitus [5].

In the past few years, rare cases of PP following bariatric surgery have been described [6, 7] The limited number of reported PP cases following bariatric surgery may be due to under-recognition and underdiagnosis. With the increasing prevalence of these procedures, it is important to raise awareness among physicians about this rare complication.

Finally, treatment of PP is multifactorial, most importantly is initiating antibacterial therapy, tetracyclines such as minocycline is the most common prescribed oral antibiotics [8]. Additionally, the need to stop the ketogenic state in the body and increase carbohydrates in the diet will help to ultimately resolve the rash [9].
